# *Cobitis laterstriata* sp. nov., a spined loach from East China (Actinopterygii, Cypriniformes, Cobitidae)

**DOI:** 10.3897/zookeys.1296.206144

**Published:** 2026-11-02

**Authors:** Xue-Yuan Li, Zhi-Xian Sun, Hao-Jun Chen, Yu-Tian Fang, Ya-Hui Zhao

**Affiliations:** 1 State Key Laboratory of Animal Biodiversity Conservation and Integrated Pest Management, Institute of Zoology, Chinese Academy of Sciences, Beijing, 100101, China State Key Laboratory of Animal Biodiversity Conservation and Integrated Pest Management, Institute of Zoology, Chinese Academy of Sciences Beijing China; 2 University of Chinese Academy of Sciences, Beijing, China University of Chinese Academy of Sciences Beijing China; 3 Shanghai Universities Key Laboratory of Marine Animal Taxonomy and Evolution, Shanghai Ocean University, Shanghai, 201306, China Shanghai Universities Key Laboratory of Marine Animal Taxonomy and Evolution, Shanghai Ocean University Shanghai China; 4 Institute of Hydrobiology, Chinese Academy of Sciences, Wuhan 430072, Hubei Province, China Institute of Hydrobiology, Chinese Academy of Sciences Wuhan China

**Keywords:** Freshwater fish, molecular phylogenetics, morphology, new species, taxonomy

## Abstract

A new spined loach, *Cobitis laterstriata*, is described from the Shuiyangjiang River (a primary tributary of the Yangtze River) in Anhui Province and the Tianmuxi River (a tributary of the Changhuaxi River) in Zhejiang Province, China. Currently, only eight species of *Cobitis* that lack sexual dimorphism and the Gambetta pattern have been recorded in China. This new species also belongs to this small group and can be distinguished from the eight known congeners by the following key characters: 1) dorsum with dense irregular cloud-like blotches, a clear blank zone above the mid-lateral line below blotches, a distinct black mid-lateral stripe from the posterior margin of the operculum to the caudal-fin base, and 10–15 irregular black blotches directly ventral; 2) lower lip separated from the mandible, and mental lobes well developed 3) short caudal peduncle; 4) 19–20 pre-pelvic myotomes; 5) dorsal fin with six branched rays, and anal fin with four branched rays. Molecular phylogenetic analyses based on the cytochrome *b* gene also confirmed the validity of this new species.

## Introduction

*Cobitis* Linnaeus, 1758, is a genus of small benthic fishes that exhibits high species diversity (Dabry de Thiersant 1872; Kottelat and Freyhof 2007; Kim 2009; Nelson et al. 2016; Freyhof et al. 2018). *Cobitis* are widely distributed across Eurasia (excluding the Tibetan Plateau), the Greater Sunda Islands, and Morocco and Abyssinia in Africa, typically inhabiting a variety of freshwater habitats, such as streams with moderate water flow and fine substrate, and lakes (Chen 1981; Sawada 1982; Bănărescu 1990). To date, a total of 130 valid species of *Cobitis* have been recorded worldwide, of which 34 occur in China (Eschmeyer et al. 2016). However, the limited morphological differentiation makes it difficult to recognize the species in this genus. Gambetta pattern—comprising five bands from dorsal to ventral (L1–L5), as established by Gambetta (1934) and Takeda and Fujie (1945)—has been widely used in its taxonomy (Gambetta 1934; Takeda and Fujie 1945; Chen and Chen 2005a; Chen 2007). The main diagnostic characteristics of *Cobitis* are as follows: eyes with a movable suborbital spine; mouth with three pairs of barbels; anal fin with four or five branched rays; in most species, five rows of Gambetta lines on both sides of the body; and swim bladder bilocular, with anterior chamber enclosed in an osseous capsule. In addition, sexual dimorphism is present in this genus, with males generally smaller than females, having longer and broader pectoral fins and a variably shaped lamina circularis at the base of the pectoral fins (Erkakan et al. 1999; Son and He 2005; Kim 2009; Chen and Chen 2011, 2013, 2016; Chen et al. 2013, 2015, 2016; Freyhof et al. 2018; Tan et al. 2019; Sun et al. 2025).

Nalbant (1963) established the genus *Niwaella*. It shares similar morphological characteristics with *Cobitis* except for the absence of the Gambetta pattern and the lack of sexual dimorphism. However, Šlechtová et al. (2008) and Perdices et al. (2016) used *Cytb* and RAG1 genes to construct phylogenetic trees for various groups within Cobitidae, and the results of both studies suggested that *Niwaella* should be considered a synonym of *Cobitis*.

In eastern China, a morphologically specialized group was previously assigned to *Niwaella* by Chen and Chen (2005b). This group comprises a total of eight species in China, including *C. brevipinna* (Chen & Chen, 2017), *C. fimbriata* (Chen & Chen, 2017), *C. laterimaculata* Yan & Zheng, 1984, *C. longibarba* (Chen & Chen, 2005b), *C. nigrolinea* (Chen & Chen, 2017), *C. qujiangensis* (Chen & Chen, 2017), *C. tigrina* (Zhang, Zhang, Chen & Zhang, 2025) and *C. xinjiangensis* (Chen & Chen, 2005b). This species group can be distinguished from other *Cobitis* species by two main characteristics mentioned above: the absence of sexual dimorphism and the lack of the Gambetta pattern. They are primarily distributed in the Tiaoxi River, Yongjiang River, Qiantang River, and the middle and lower reaches of the Yangtze River in Anhui, Zhejiang, and Jiangxi Provinces.

During the examination of specimens collected from the Shuiyangjiang River, a primary tributary of the Yangtze River in Ningguo City, Anhui Province and the Tianmuxi River, a tributary of the Changhuaxi River, Linan District, Hangzhou City, Zhejiang Province, we recognized a new *Cobitis* species, which should be assigned to the group characterized by the absence of both sexual dimorphism and the Gambetta pattern. Integrative evidence from morphological comparisons and molecular phylogenetic analyses confirms that it represents an undescribed taxon within *Cobitis*.

## Material and methods

### Species collection, examination, and preservation

**Figure 1.**
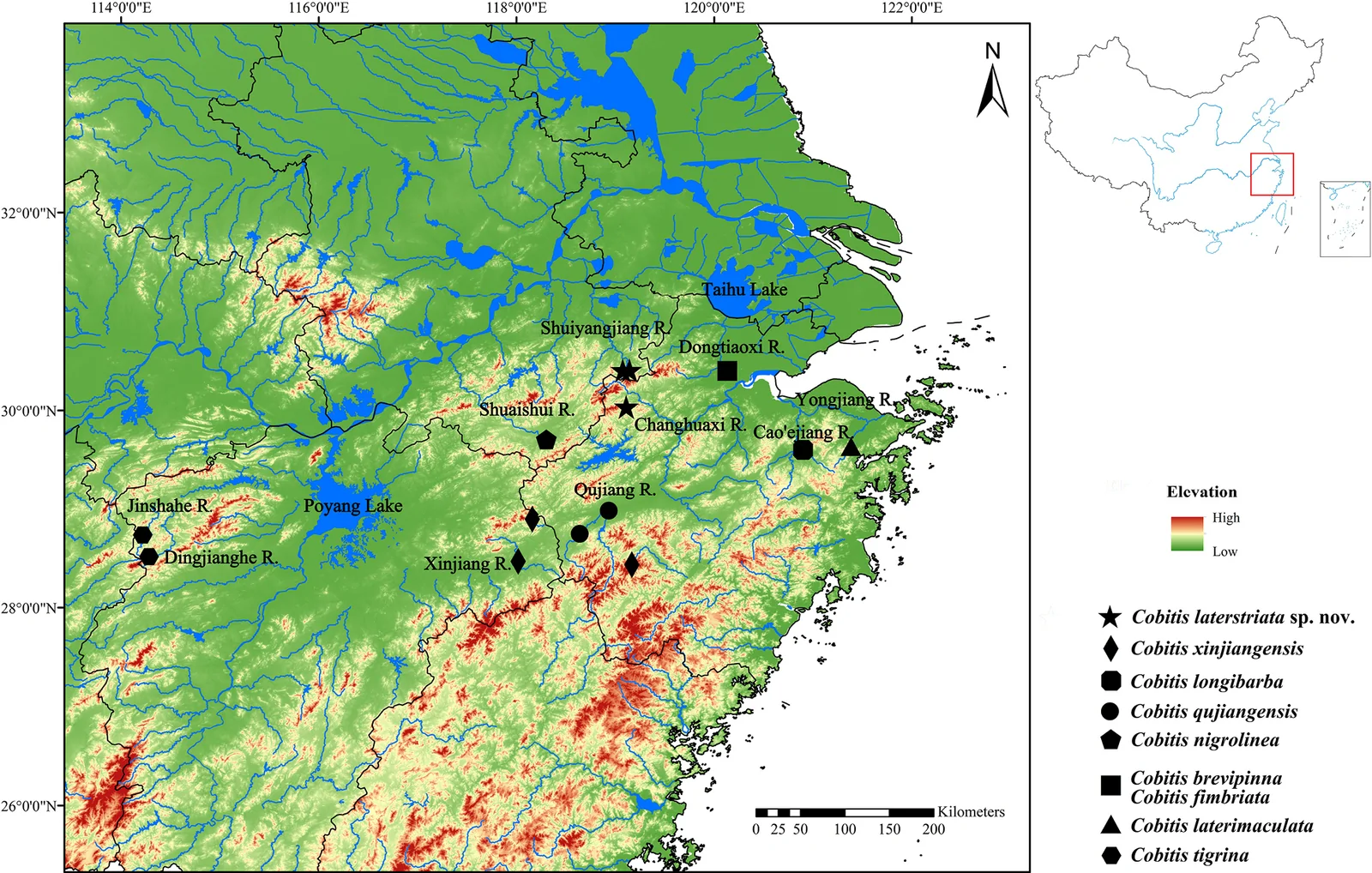
Localities of the nine species within *Cobitis* in China, which both lack sexual dimorphism. The distribution information for the other eight species was compiled from Yan and Zheng (1984), Chen and Chen (2005b), Chen et al. (2017), and Zhang et al. (2025).

The type specimens were collected from a tributary of the Shuiyangjiang River, Wanjia Township, Ningguo City, Anhui Province (30.4045°N, 119.1441°E, 130 m a.s.l.). The specific collection localities of the other specimens of the new species are shown in Fig. 1 and the Results section. All holotype and paratype specimens are deposited at the Institute of Zoology, Chinese Academy of Sciences, Beijing, China (ASIZB; abbreviation follows Sabaj 2020). All specimens used in this study were handled in compliance with China’s regulations on laboratory animal welfare and ethical review. All examined specimens (*N* = 108) were collected using hand nets or fish traps, or purchased from local markets (see Comparative Materials for detailed information). Specimens intended for morphological analysis were initially fixed in 10% formalin solution for three days and subsequently transferred to 70% ethanol for long-term preservation. Specimens designated for molecular phylogenetic studies were immediately preserved in 95% ethanol.

### Morphological study

Only specimens with intact morphology were selected for morphological analysis. Meristic counts included the number of branched rays in dorsal, anal, pectoral, pelvic, and caudal fins, the number of pre-pelvic myotomes, and the total number of vertebrae. The last full or half ray in dorsal and anal fins was counted as one. Pre-pelvic myotome counts were defined as the number of segmented muscle plates between the base of the pectoral fin and the insertion of the pelvic fin (Nakajima and Suzawa 2016). Gambetta pattern: The longitudinal five rows of color bands along the body, designated L1–L5 sequentially from dorsal midline to ventral side. Specifically, L1 is typically saddle-shaped black blotches; L2 usually consists of small blotches that occasionally confluent with L1; L3 is generally composed of larger rounded black spots, which may sometimes fuse and even connect into a continuous band; L4 is commonly made up of densely arranged tiny black dots, and these dots can also merge into a band in some cases; L5 is typically a two-layered pattern, the inner layer is a black band, and the surface layer is covered with regularly arranged large black patches (Gambetta 1934; Takeda and Fujie 1945; Chen and Chen 2005a; Chen 2007). All meristic counts were conducted on the left side of specimens. If structures on the left side were damaged, the corresponding structures on the right side were counted instead. X-ray images were obtained using an XDR-AZ1600 X-ray inspection system (Shanghai Anzhu Optoelectronics Co., Ltd) with a tube voltage of 60 kV, tube current of 1 mA, and exposure time of 2 s. Vertebral counts were based on these radiographs. The Weberian apparatus posterior to the skull was counted as four vertebrae, and the last half vertebra near the base of the caudal fin was counted as one. The total number of vertebrae was expressed as “4 + n”, where n represents the number of vertebrae excluding the Weberian apparatus.

Measurable traits were precisely measured on the left side of each specimen using a digital caliper with an accuracy of 0.01 mm. All measurements were taken point-to-point following standard protocols. The schematic diagrams for the measurement standards of the measurable traits (e.g., standard length SL, head length HL) used in this study are referenced from Sun et al. (2025). Detailed morphometric data are presented in Table 2.

Additional features, such as scale morphology and suborbital spines, were observed and photographed under a stereomicroscope. Photographs of fish specimens were taken using a digital single-lens reflex camera.

Principal component analysis (PCA) was used to further explore morphological variation. A total of 24 morphological measurements, including standard length, body depth, and head length, were used for the PCA. Data were log10-transformed prior to analysis and processed using PAST v. 4.17) (Hammer et al. 2001).

Mann-Whitney U tests were conducted using R 4.3.3 (Ihaka and Gentleman 1996). The significance level was set at p < 0.05. For each pairwise comparison, the U statistic and corresponding p-value were calculated. A p-value of less than 0.05 was considered statistically significant.

### Molecular phylogenetic analyses

In this study, the mitochondrial cytochrome *b* (*Cytb*) gene was selected as molecular marker for phylogenetic tree reconstruction and elucidation of evolutionary relationships among species. The right pelvic fin of fish specimens was preserved in 95% ethanol. Mitochondrial DNA was extracted using a standard phenol-chloroform protocol. The *Cytb* was amplified with the universal primers L14724 (5'-GACTTGAAAAACCACCGTTG-3') and H15915 (5'-CTCCGATCTCCGGATTACAAGAC-3'). PCR was performed as follows: pre-denaturation at 94 °C for 5 min, followed by 30 s denaturation at 94 °C, 30 s annealing at 58 °C, and 30 s extension at 72 °C for 36 cycles, then final extension at 72 °C for 7 min. Sequencing reactions were performed following the BigDye Terminator v. 3.1 (BDT) protocol, with a total reaction volume of 5 µL containing 1 µL primer (3.2 pmol/µL), 1 µL template DNA, 0.4 µL BigDye Terminator v. 3.1, 0.8 µL sequencing buffer and 1.8 µL double-distilled water (ddH_2_O). The thermal cycling conditions were as follows: initial denaturation at 94 °C for 5 min; 25 cycles of denaturation at 94 °C for 10 s, annealing at 55 °C for 5 s, and extension at 60 °C for 90 s. The sequencing products were stored at 4 °C. All of the above procedures, including extraction, PCR amplification, and sequencing, were performed by Beijing Tianyi Huiyuan Biotechnology Co., Ltd using Sanger sequencing.

Sequences from the company were assembled using SeqMan. Other sequences used in this study were acquired from NCBI (https://www.ncbi.nlm.nih.gov/). The voucher ID for each sequence and GenBank accession number for the *Cytb* gene are listed in Table 1. The assembled sequences, together with those retrieved from NCBI, were imported into PhyloSuite v. 1.2.3 in FASTA format for phylogenetic reconstruction. All sequences were aligned using MAFFT. An aligned fragment of 1140 bp of the *Cytb* gene was extracted by trimming the non-target regions at both ends of the aligned sequences. The best-fit substitution model was selected using ModelFinder under the Bayesian Information Criterion (BIC) (Kalyaanamoorthy et al. 2017). A Bayesian inference (BI) tree was constructed using MrBayes v. 3.2.7 under the HKY+F+G4 model (Ronquist et al. 2012). Two parallel runs of Markov chain Monte Carlo (MCMC) simulations were performed for 2,000,000 generations, with the first 25% of samples discarded as burn-in. A maximum likelihood (ML) tree was constructed using IQ-TREE v. 2.2.0 under the HKY+F+G4 model. Branch support was evaluated with 10, 000 bootstrapping replications, and the SH-aLRT test with 1000 replicates.

**Table 1.** Voucher codes, sampling localities and GenBank accession numbers of the *Cytb* gene for molecular analyses in the present study.

**Species**	**Voucher codes**	**Localities**	**Drainage**	**Accession no**.	**Source**
*Cobitis laterstriata*	ASIZB 254933	Ningguo City, Anhui, China	Shuiyangjiang River	-	This study
*Cobitis laterstriata*	ASIZB 254957	Ningguo City, Anhui, China	Shuiyangjiang River	-	This study
*Cobitis laterstriata*	ASIZB 254958	Ningguo City, Anhui, China	Shuiyangjiang River	-	This study
*Cobitis laterstriata*	ASIZB 254959	Ningguo City, Anhui, China	Shuiyangjiang River	-	This study
*Cobitis laterstriata*	ASIZB 254960	Ningguo City, Anhui, China	Shuiyangjiang River	-	This study
*Cobitis laterstriata*	ASIZB 254961	Ningguo City, Anhui, China	Shuiyangjiang River	-	This study
*Cobitis laterstriata*	ASIZB 254962	Ningguo City, Anhui, China	Shuiyangjiang River	-	This study
*Cobitis laterstriata*	ASIZB 254968	Hangzhou City, Zhejiang, China	Tianmuxi River	-	This study
*Cobitis laterstriata*	ASIZB 254969	Hangzhou City, Zhejiang, China	Tianmuxi River	-	This study
*Cobitis brevipinna*	ASIZB 254963	Hangzhou City, Zhejiang, China	Tianmuxi River	-	This study
*Cobitis brevipinna*	ASIZB 254964	Hangzhou City, Zhejiang, China	Tianmuxi River	-	This study
*Cobitis brevipinna*	-	Zhejiang, China	Dongtiaoxi River	KY408023	Chen et al. 2017
*Cobitis brevipinna*	-	Zhejiang, China	Dongtiaoxi River	KY408024	Chen et al. 2017
*Cobitis laterimaculata*	-	Zhejiang, China	Yongjiang River	KY408025	Chen et al. 2017
*Cobitis fimbriata*	-	Zhejiang, China	Dongtiaoxi River	KY408021	Chen et al. 2017
*Cobitis fimbriata*	-	Zhejiang, China	Dongtiaoxi River	KY408022	Chen et al. 2017
*Cobitis nigrolinea*	ASIZB 254965	Xiuning County, Huangshan City, Anhui, China	Xinanjiang River	-	This study
*Cobitis nigrolinea*	ASIZB 254966	Xiuning County, Huangshan City, Anhui, China	Xinanjiang River	-	This study
*Cobitis nigrolinea*	-	Anhui, China	Shuaishui River	KY408012	Chen et al. 2017
*Cobitis qujiangensis*	-	Zhejiang, China	Qujiang River	KY408015	Chen et al. 2017
*Cobitis qujiangensis*	-	Zhejiang, China	Qujiang River	KY408016	Chen et al. 2017
*Cobitis qujiangensis*	-	Zhejiang, China	Qujiang River	KY408017	Chen et al. 2017
*Cobitis tigrina*	-	Jiangxi, China	Xiushui River	PV365185	Zhang et al. 2025
*Cobitis longibarba*	-	Zhejiang, China	Cao’ejiang River	KY408026	Chen et al. 2017
*Cobitis longibarba*	-	Zhejiang, China	Cao’ejiang River	KY408027	Chen et al. 2017
*Cobitis xinjiangensis*	-	Jiangxi, China	Xinjiang River	KY408011	Chen et al. 2017
*Cobitis xinjiangensis*	ASIZB 254999	Jiangxi, China	Xinjiang River	-	This study
*Cobitis xinjiangensis*	ASIZB 255000	Jiangxi, China	Xinjiang River	-	This study
*Cobitis xinjiangensis*	ASIZB 255002	Jiangxi, China	Xinjiang River	-	This study
*Cobitis xinjiangensis*	ASIZB 255003	Jiangxi, China	Xinjiang River	-	This study
*Cobitis delicata*	-	Gifu County, Japan	Takahara River	AP009308	Saitoh et al. 2010
*Cobitis delicata*	-	Japan	-	AP011230	Miya 2009 (Direct Submission)
*Cobitis delicata*	-	Gifu County, Japan	Takahara River	NC015307	Saitoh et al. 2010
*Cobitis crassicauda*	-	Yujiang County, Jiangxi, China	Xinjiang River	JX888909	Chen and Chen 2013
*Cobitis fasciola*	-	Yujiang County, Jiangxi, China	Xinjiang River	JX888910	Chen and Chen 2013
*Cobitis stenocauda*	-	Jiangxi, China	Xinjiang River	JX888903	Chen and Chen 2013
*Cobitis zhejiangensis*	ASIZB 229984	Jingyun County, Lishui City, Zhejiang, China	Zhenxi River	-	This study
*Cobitis dolichorhynchus*	ASIZB 254967	Zhangping City, Fujian, China	Jiulongjiang River	-	This study
*Sabanejewia aurata*	-	Georgia	Rioni River	AF499190	Perdices et al. 2003

The resulting phylogenetic trees were uploaded to the TVBOT website (https://www.chiplot.online/tvbot.html, Xie et al. 2023) for visualization. The root was reassigned using *Sabanejewia aurata* (De Filippi, 1863) as the outgroup, branch lengths were omitted, and bootstrap values were displayed at each node.

Genetic distances within and between species were calculated based on the Kimura two-parameter model.

## Results

### Cobitis laterstriata

Taxon classificationAnimaliaCypriniformesCobitidae

Li & Zhao sp. nov.

A66567DB-3AAE-5632-8483-28BA4B5F2F67

https://zoobank.org/A882B185-A892-4E91-A054-8208E9C910C6

**Figure 2.**
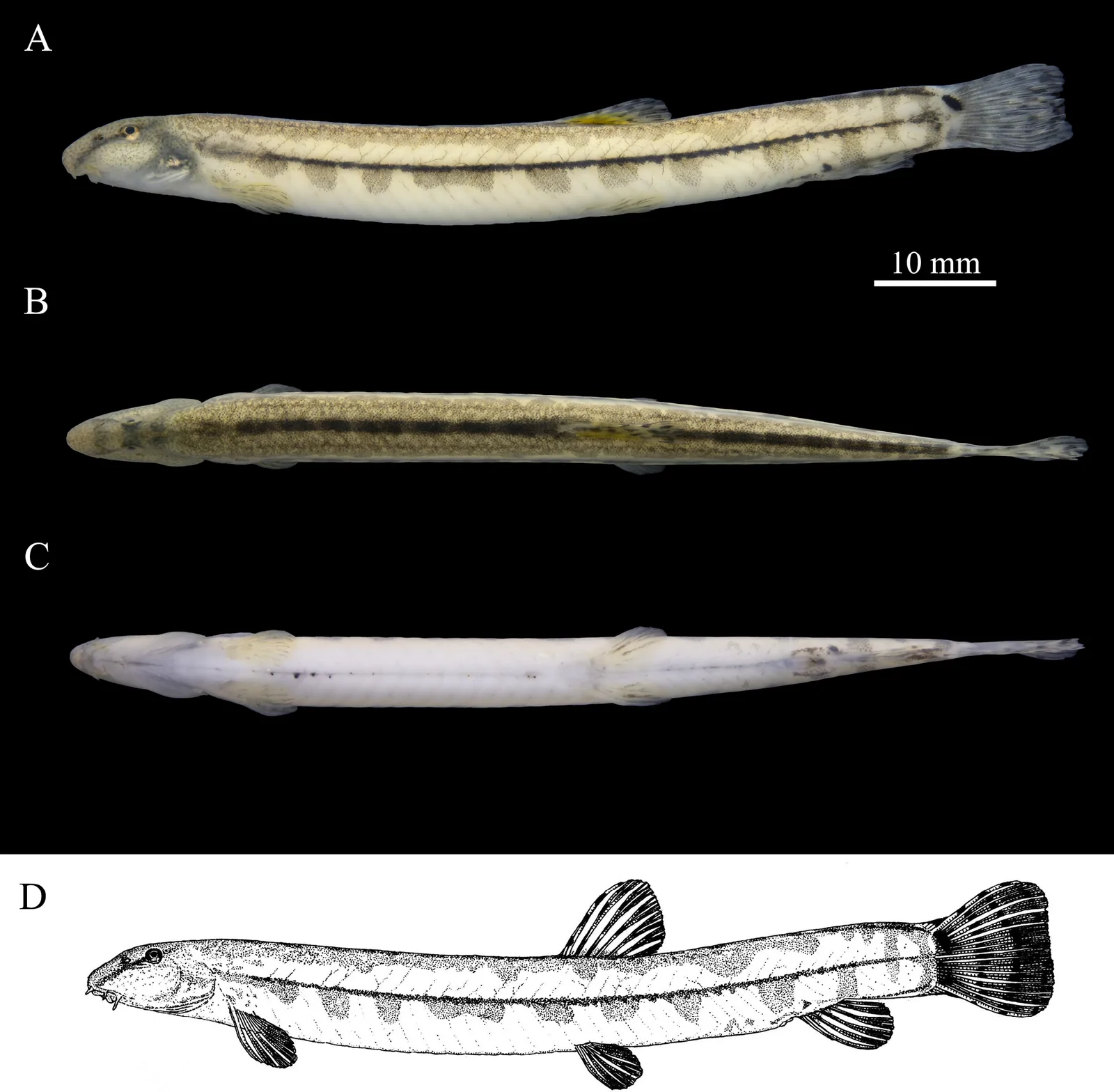
*Cobitis laterstriata* sp. nov., holotype, ASIZB 254919, 71.7 mm SL. **A**. Lateral view; **B**. Dorsal view; **C**. Ventral view; **D**. Hand drawing.

**Figure 3.**
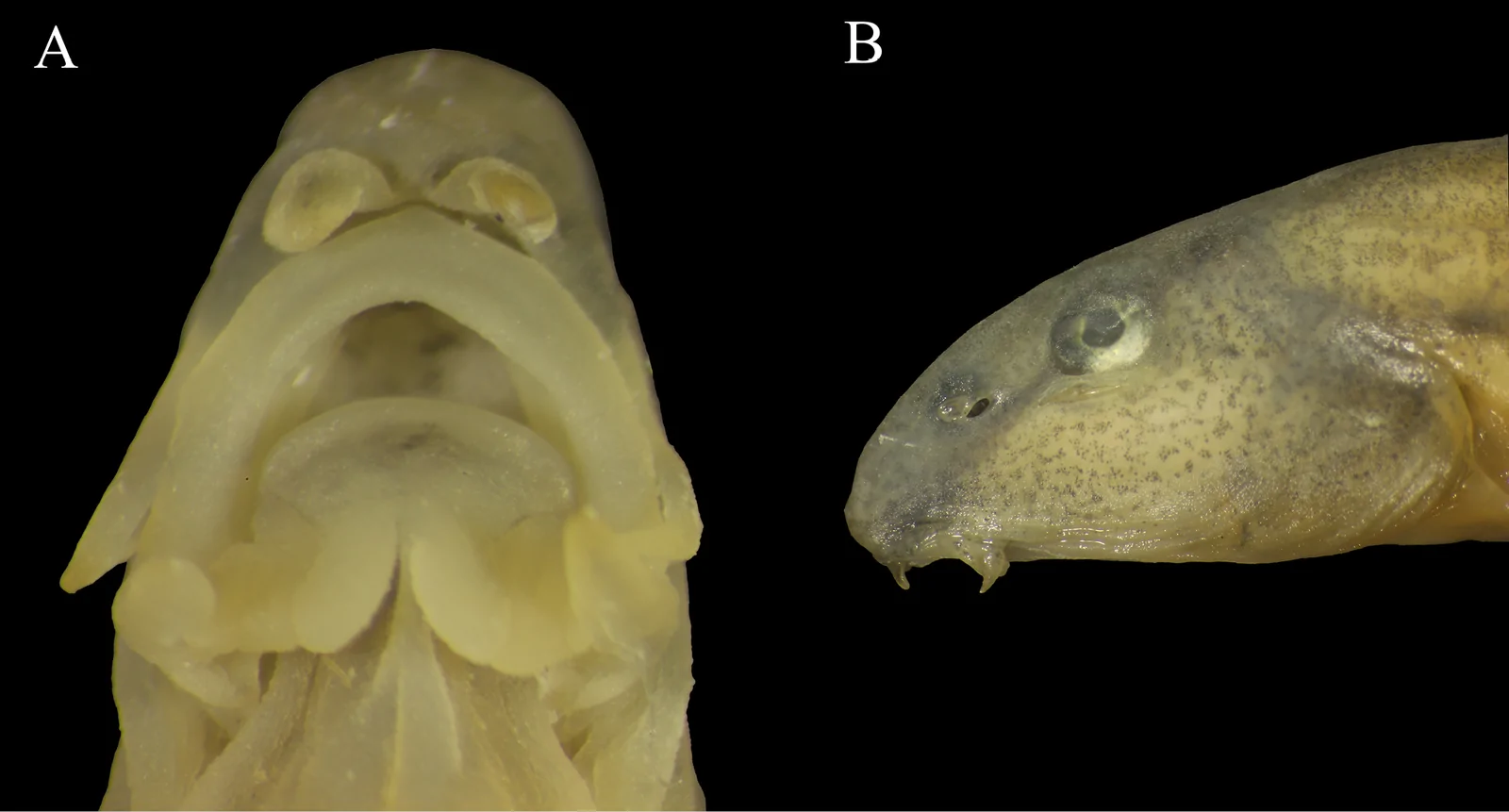
*Cobitis laterstriata* sp. nov., ASIZB 254937. **A**. Mouth, ventral view; **B**. Lateral view of head.

**Figure 4.**
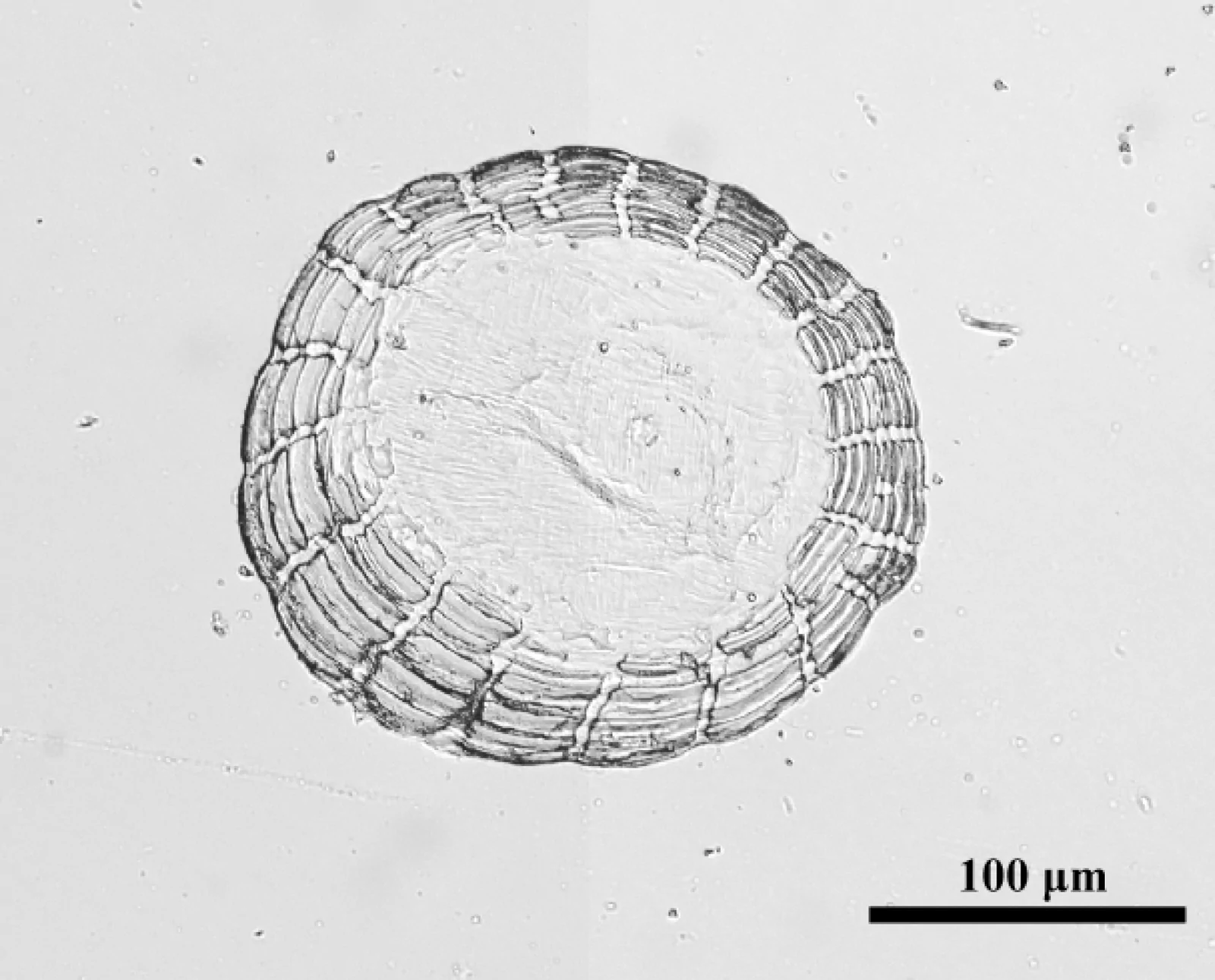
Subdorsal scales of *Cobitis laterstriata* sp. nov.

**Figure 5.**
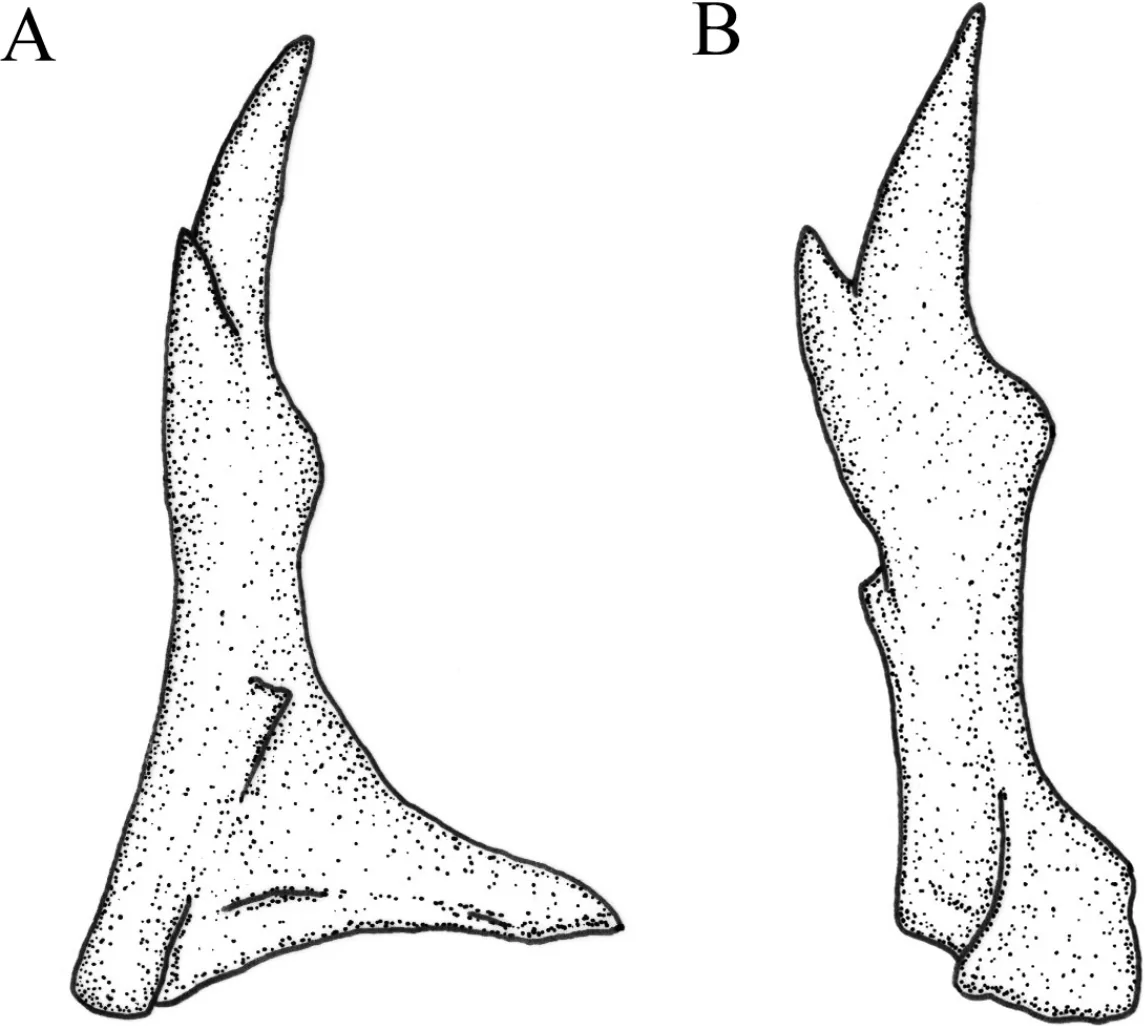
Right suborbital spine of *Cobitis laterstriata* sp. nov. **A**. Lateral view; **B**. Dorsal view.

Figs 2, 3, 4, 5, Table 2

#### Type material.

***Holotype***. • ASIZB 254919, 71.7 mm SL; Wanjia Township, Ningguo City, Anhui Province, from a tributary of the Shuiyangjiang River, which is a tributary of the Yangtze River (30.4045°N, 119.1441°E, 130 m a. s. l.); collected by Haojun Chen; August 21, 2025. ***Paratypes***. 47 specimens • ASIZB 254918, 254920, 254934–254962, 254970–254985, 40.8–74.3 mm SL; same collection information as holotype.

#### Diagnosis.

The new species can be distinguished by the following combination of characters: no Gambetta pattern; dorsal blotches barely extend to lateral side; upper lateral side covered with dense, irregular cloud-shaped blotches; a blank zone above mid-lateral line ventral to these blotches; a well-defined black mid-lateral stripe extending from posterior edge of operculum to caudal-fin base, with 10–15 irregular black blotches scattered directly ventrally; lower lip and mandible separated, with well-developed mental lobes; caudal peduncle short; 19–20 pre-pelvic myotomes; dorsal fin with six branched rays; anal fin with four branched rays.

#### Description.

Body medium-sized, elongated, slightly compressed laterally. Body width and depth tapering gently posterior to dorsal fin toward caudal-fin base. Head ventral surface flattened; abdomen slightly rounded; dorsal body profile smoothly ascending snout-occiput; occiput to caudal-fin base nearly horizontal. Caudal peduncle short, laterally compressed, with developed fleshy keels on dorsal and ventral side. Head short, small, laterally compressed. Snout bluntly rounded (lateral view), sharply triangular (dorsal view); snout length subequal to postorbital length. Eye small, superior and laterally positioned, with convex and narrow interorbital space. Anterior and posterior nostrils closely situated; anterior nostril with short tube. Anus located immediately anterior to anal-fin insertion (Figs 2, 3, Table 2).

**Table 2.** Counts and measurements of *Cobitis laterstriata* sp. nov., *C. xinjiangensis* and *C. nigrolinea*.

**Characteristics**	***Cobitis laterstriata* sp. nov**.			***Cobitis xinjiangensis***		***Cobitis nigrolinea***	
	Holotype (H)	Paratypes + H (*N* = 48)		(*N* = 16)		(*N* = 16)	
		Mode (range)	n	Mode (range)	n	Mode (range)	n
Branched dorsal-fin rays	6	6	48	6	16	7	10
Brached anal-fin rays	4	4	48	5	16	5	10
Branched pectoral-fin rays	7	7	48	7	16	7	10
Branched pelvic-fin rays	6	6	48	6	16	6	10
Branched caudal-fin rays	14	14	48	14	16	14	10
Pre-pelvic myotomes	19	19 (19–20)	36	17 (17–18)	14	18 (17–18)	9
Vertebrae	4+42	4+42 (42–44)	8	4+42 (42–43)	5	4+42 (41–42)	5
		Mean (range)	SD	Mean (range)	SD	Mean (range)	SD
Standard length (mm)	71.7	55.5 (40.8–74.3)	-	53.9 (42.4–74.6)	-	61.0 (51.7–78.3)	-
**In percentage of SL %**							
Body depth	10.4	11.4 (9.4–13.0)	0.7	12.7 (11.8–13.9)	0.6	10.5 (9.1–11.4)	0.7
Head length	15.6	16.1 (14.5–18.2)	0.8	18.2 (16.6–19.3)	0.8	17.2 (15.9–19.2)	1.0
Dorsal-fin length	14.0	13.1 (11.5–16.0)	0.8	15.0 (13.7–15.9)	0.6	15.1 (13.9–16.8)	0.8
Dorsal-fin base length	7.7	7.3 (5.6–9.8)	0.8	9.2 (8.4–10.9)	0.7	8.5 (7.5–9.7)	0.6
Pectoral-fin length	9.3	9.1 (7.6–10.5)	0.7	10.4 (9.2–12.0)	0.8	10.1 (9.4–11.9)	0.6
Pectoral-fin base length	1.4	2.1 (1.4–2.9)	0.3	2.1 (1.6–2.6)	0.3	2.1 (1.6–2.6)	0.3
Pelvic-fin length	9.1	7.9 (6.3–9.3)	0.6	9.1(8.5–10.2)	0.5	9.0 (8.0–10.2)	0.5
Pelvic-fin base length	1.6	2.2 (1.6–2.9)	0.3	2.2 (1.6–2.9)	0.3	2.1 (1.6–2.6)	0.3
Anal-fin length	10.0	9.8 (7.6–12.8)	1.0	11.3 (12.9–10.1)	0.8	11.3 (10.3–12.0)	0.5
Anal-fin base length	4.2	4.5 (3.4–5.9)	0.6	6.2 (5.7–7.0)	0.3	5.7 (5.0–6.9)	0.5
Caudal peduncle length	10.0	9.8 (8.2–11.3)	0.6	13.9 (11.4–16.9)	1.2	12.5 (10.6–14.5)	1.1
Caudal peduncle depth	7.2	7.8 (6.1–9.0)	0.6	8.3 (7.0–9.4)	0.6	7.8 (7.1–8.8)	0.4
Pre-dorsal length	56.4	58.4 (55.8–62.2)	1.5	55.4 (52.9–58.7)	1.6	56.2 (52.2–58.8)	1.5
Pre-pectoral length	17.1	18.2 (16.1–20.6)	1.0	19.7 (17.6–21.2)	1.1	19.2 (17.5–20.7)	0.9
Pre-pelvic length	58.5	60.1 (55.8–63.0)	1.2	54.9 (52.0–59.6)	1.9	56.2 (54.4–58.8)	1.1
Pre-anal length	86.8	85.8 (78.0–89.5)	1.9	79.1 (74.5–83.0)	2.2	82.0 (80.0–84.1)	1.4
Post-dorsal length	44.8	42.4 (39.1–46.2)	1.3	47.0 (44.3–48.9)	1.3	45.6 (43.7–47.9)	1.2
Distance between pectoral -fin and pelvic-fin insertion	42.1	42.3 (39.1–46.1)	1.3	35.4 (31.3–39.3)	2.1	37.5 (35.2–39.6)	1.4
**In percentage of HL %**							
Head depth	49.6	55.2 (47.8–60.5)	2.9	55.3 (46.1–60.6)	3.7	53.6 (49.7–57.8)	2.1
Head width	38.1	44.5 (35.9–55.3)	3.6	44.0 (37.6–52.0)	4.2	43.0 (37.4–47.4)	2.5
Eye diameter	12.1	13.4 (10.6–17.5)	1.5	12.9 (10.1–14.9)	1.4	13.8 (10.9–16.0)	1.3
Interorbital width	8.2	11.1 (8.2–14.2)	1.6	12.3 (8.5–20.5)	2.8	10.9 (9.0–13.2)	1.1
Snout length	44.0	42.8 (34.6–47.6)	2.4	45.2 (41.8–50.4)	2.5	45.6 (42.3–47.6)	1.3

Mouth small, inferior. Three pairs of short barbels: one pair of rostral barbels not reaching mouth corners; one pair of maxillary barbels not reaching under anterior edge of orbit; one pair of maxillomandibular barbels shorter than or almost equal to eye diameter. Lips fleshy, smooth; upper lip broad, without affiliated apparatus; lower lip short, separated from mandible, divided into two well-developed mental lobes (Fig. 3).

Head scaleless. Body covered with small, round or oval scales. Each scale has a large eccentric focal area (being closer to base), with 14–24 radial grooves, and one to five supplementary radial grooves (radial grooves broad, sparse anteriorly, dense basally) (Fig. 4). Lateral line short, not reaching to tip of pectoral fin. Pre-pelvic myotomes 19 or 20 (Table 2).

Dorsal fin short, with three unbranched and six branched rays, located in posterior half of body; distal margin slightly convex. Pectoral fins short, with one unbranched and seven branched rays. Pelvic fins with one unbranched and six branched rays, inserted below second or third branched dorsal-fin ray. Anal fin short and small, with three unbranched and four branched rays, inserted nearer to caudal-fin base than to pelvic-fin insertion. Caudal fin slightly truncated, with one unbranched principal ray and seven branched principal rays on upper part and seven branched principal rays and one unbranched principal ray on lower part. (Fig. 2, Table 2).

Total vertebrae 4+42 (4 specimens), 43 (2), 44 (2) (Table 2). Suborbital spine thick and curved; tip reaching middle of orbit (Fig. 5).

#### Coloration in live specimens.

Dorsal and lateral surfaces of head and body light brownish-yellow; ventral surface grayish white; operculum with slight iridescence. A distinct dark black stripe extends from insertion of first rostral barbel, passing through eye, to occiput. Numerous dense black spots on head, sometimes forming several black stripes under eye. No Gambetta pattern. A conspicuous black stripe along dorsal midline, extending from occiput, passing through dorsal fin, to caudal-fin base, with 14–19 uniformly spaced, small, short black blotches embedded in stripe, which barely extend to lateral side; irregular, dense, cloud-like black plaques on upper lateral side (smaller, denser post-head; larger, scattered toward caudal peduncle); a blank zone above mid-lateral line ventral to these blotches; almost devoid of blotches. A prominent, dark black fine stripe along mid-lateral line, extending from posterior margin of operculum to caudal-fin base; 10–15 irregular black blotches closely arranged ventral to mid-lateral line (oblong-oval anteriorly, vertically elongated posteriorly); sometimes coalescing into a broad black stripe in anterior body which may be sex-related. All fins hyaline and whitish; except pectoral and pelvic-fins base pale light orange. Three to four rows of black spots present on dorsal and caudal-fin rays; few black spots on distal part of pectoral fin and base of pelvic fin and anal fin. A distinct, jet-black large spot on upper caudal-fin base; a smaller, paler spot sometimes present ventrally to it (Fig. 10).

#### Coloration in preserved specimens.

Dorsal and lateral surfaces of head and body light yellow; ventral surface pale; operculum without slight iridescence. All patterns in same position as living specimen (Fig. 2).

#### Sexual dimorphism.

Not obvious in external morphology.

#### Distribution and habitat.

**Figure 6.**
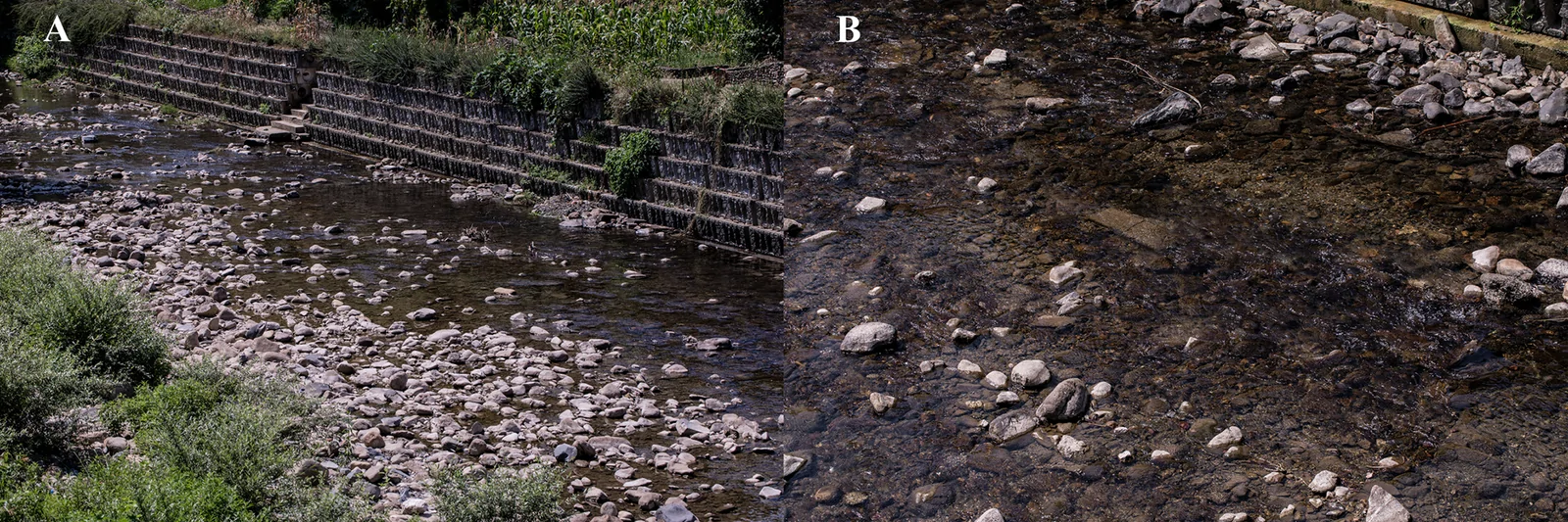
Natural habitats at the type locality of *Cobitis laterstriata* sp. nov. **A**. Habitat overview; **B**. Close-up of riverbed substrate.

*Cobitis laterstriata* sp. nov. is distributed in tributaries of the Shuiyangjiang River (a tributary of the Yangtze River) in Wanjia and Nanji Township, Ningguo City, Anhui Province. This species is also found in the Tianmuxi River (a tributary of the Changhuaxi River) in Zhangjia Village, Chakou Township, Lin’an District, Hangzhou City, Zhejiang Province. This species typically inhabits shallow rivers with gravel bottoms (Figs 1, 6).

#### Etymology.

This specific name derives from the Latin *later*, meaning alongside of body, and Latin *striata* meaning stripes or bands, in reference to the prominent, dark black stripe that runs along the mid-lateral line from the posterior edge of the operculum to the caudal-fin base. The Chinese name suggested for this species is “侧带花鳅”.

##### Morphological comparison

**Figure 7.**
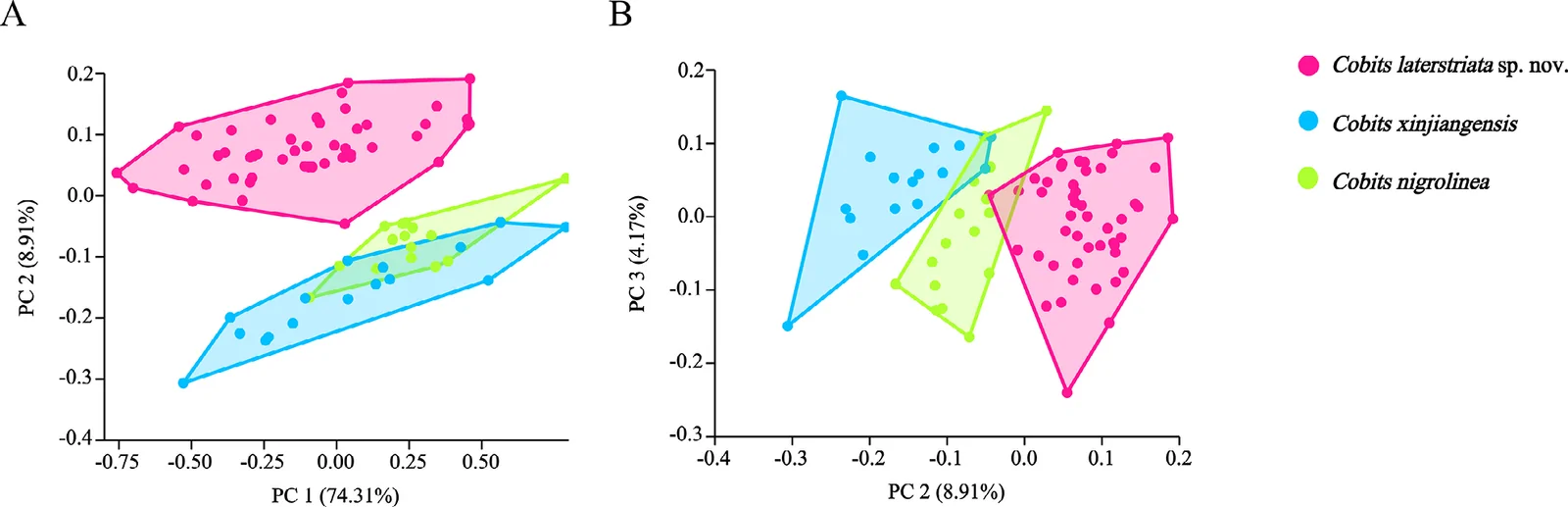
Scatterplot of PC1 against PC2 (**A**) and PC2 against PC3 (**B**).

Based on morphological measurement data, our study conducted a principal component analysis (PCA) to understand the potential morphological differences among *Cobitis laterstriata*, *C. xinjiangensis* and *C. nigrolinea*. Among these, *C. xinjiangensis* is the most morphologically similar to *C. laterstriata*, while *C. nigrolinea* shows the closest geographic proximity. The analysis incorporated 24 morphometric measurements from 48 individuals of *C. laterstriata*, 18 of *C. nigrolinea* and 16 of *C. xinjiangensis*. Results revealed that, in terms of morphology, *C. laterstriata* is significantly different from those of the other two species. The first three components contributed a total of 87.39% of the variance. Principal component 1 (PC 1) represents most of the body size of the specimens, while PC2 and PC3 represents shape-related morphological traits. The PCA loadings are presented in Table 3, showing that interorbital width, fin base length, caudal peduncle length and distance between pectoral -fin and pelvic-fin insertion contribute most to PC2 and PC3. In conclusion, both PC1 against PC2 and PC2 against PC3 are able to separate *C. laterstriata* from the other three *Cobitis* species (Fig. 7).

**Table 3.** Loadings on the first three principal components extracted from morphometric data of *Cobitis laterstriata* sp. nov., *C. xinjiangensis* and *C. nigrolinea*. Values in bold indicate higher contributions within each PC column of the PCA.

**Morphometric measurements**	**PC 1**	**PC 2**	**PC 3**
Standard length (mm)	0.1949	0.1529	-0.15578
Body depth	0.15815	0.070313	0.09568
Head length	0.18902	-0.02936	0.003096
Head depth	0.179	-0.01386	0.083642
Head width	0.21274	0.053632	0.085601
Dorsal-fin length	0.22676	-0.11272	-0.17275
Dorsal-fin base length	0.24669	-0.22109	-0.19183
Pectoral-fin length	0.22654	-0.04359	-0.09873
Pectoral-fin base length	0.22363	0.27413	**0.5208**
Pelvic-fin length	0.22088	-0.09636	-0.09724
Pelvic-fin base length	0.16558	0.24044	**0.35933**
Anal-fin length	0.2234	-0.13239	-0.06648
Anal-fin base length	0.22922	**-0.42124**	0.004645
Caudal peduncle length	0.27877	**-0.34518**	0.007685
Caudal peduncle depth	0.18802	0.09449	0.001603
Eye diameter	0.14821	-0.03213	0.03417
Interorbital width	0.19898	-0.10955	**0.54866**
Snout length	0.23184	-0.09525	0.017941
Pre-dorsal length	0.17918	0.23475	-0.13287
Pre-pectoral length	0.16902	-0.00565	-0.07273
Pre-pelvic length	0.17403	0.29709	-0.17669
Pre-anal length	0.18229	0.28207	-0.17633
Post-dorsal length	0.2229	-0.00319	-0.1494
Distance between pectoral -fin and pelvic-fin insertion	0.17161	**0.44032**	-0.22681

##### Molecular phylogeny and genetic comparisons

A total of 38 sequences from 15 *Cobitis* species were included in the analyses. Among these, a total of 19 new *Cytb* sequences were obtained through our field surveys, including nine *Cytb* sequences of new species, two sequences of *C. brevipinna*, two sequences of *C. nigrolinea*, four sequences of *C. xinjiangensis*, one sequence of *C. zhejiangensis* Son & He, 2005 and one sequence of *C. dolichorhynchus* Nichols, 1918. Additional sequences downloaded from NCBI including two *Cytb* sequences from *C. brevipinna*, one sequence from *C. laterimaculata*, two sequences from *C. fimbriata*, one sequence from *C. nigrolinea*, three sequences from *C. qujiangensis*, one sequence from *C. tigrina*, two sequences from *C. longibarba*, one sequence from *C. xinjiangensis*, three sequences from *C. delicata* Niwa, 1937, one sequence from *C. crassicauda* Chen & Chen, 2013, one sequence from *C. fasciola* Chen & Chen, 2013 and one sequence from *C. stenocauda* Chen & Chen, 2013. One sequence from *Sabanejewia aurata* was designated as the outgroup.

**Figure 8.**
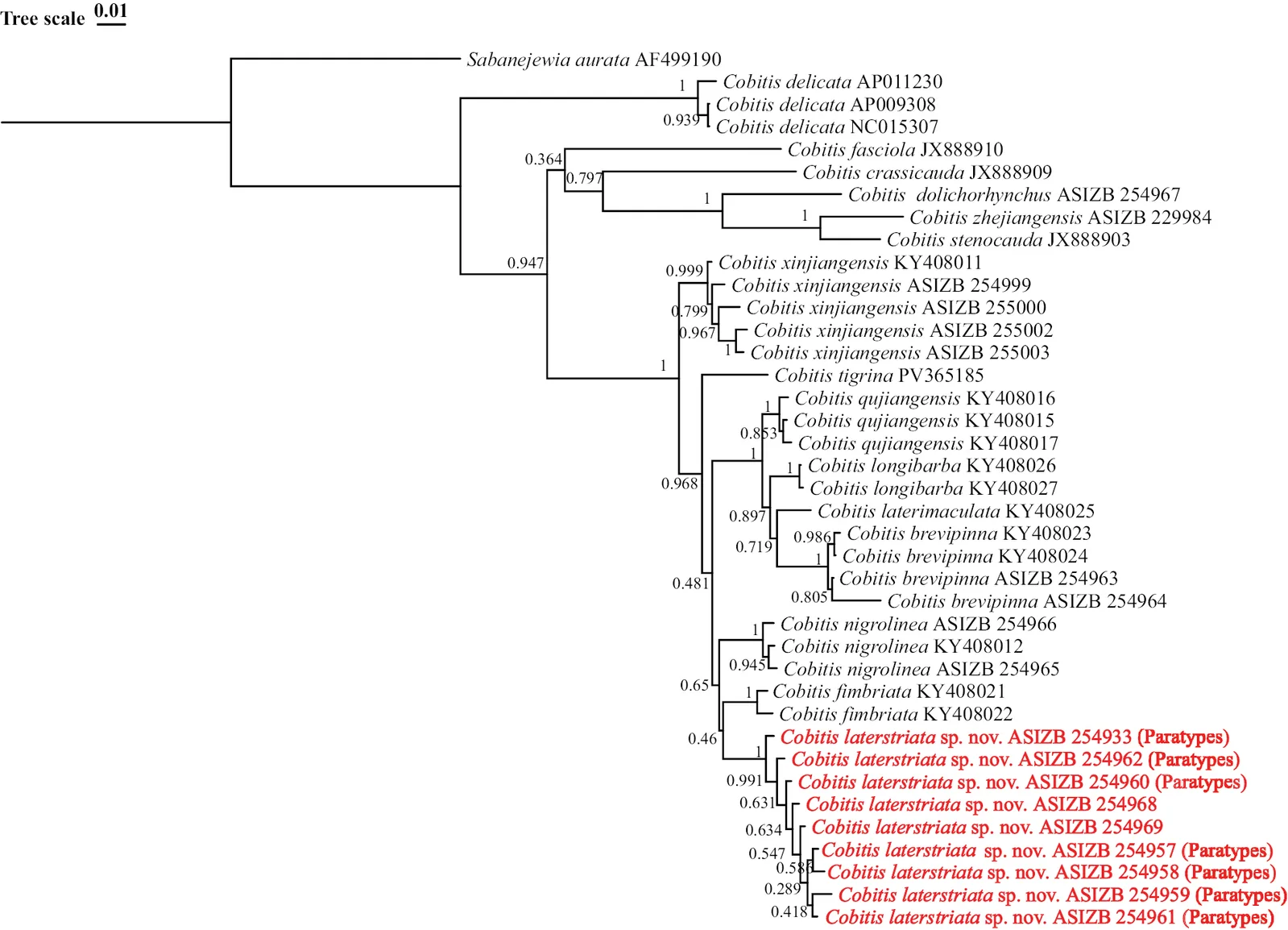
Bayesian tree of the new species and its close relatives recovered from mitochondrial cytochrome *b* based on the HKY+F+G4 model. Bayesian posterior probabilities are shown at the nodes.

**Figure 9.**
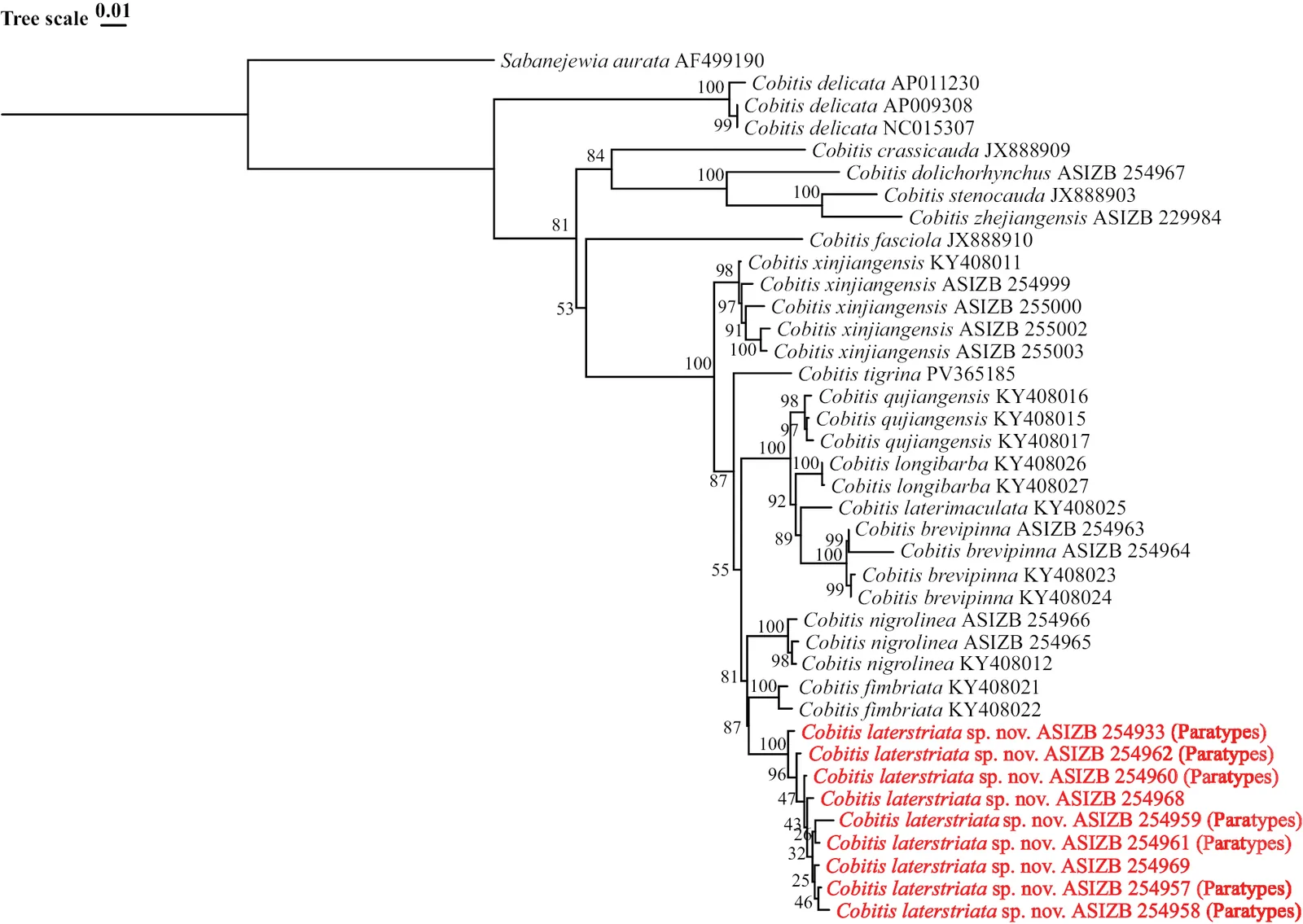
Maximum likelihood tree of the new species and its close relatives inferred from mitochondrial cytochrome *b* gene sequences based on the HKY+F+G4 model. Bootstrap support values are shown at nodes.

The molecular phylogenetic results (BI and ML trees in Figs 8, 9) based on mitochondrial *Cytb* sequences show that nine samples of the newly described species form a monophyletic group itself, which is a sister group to *C. fimbriata*. These samples were also closely related to those of other Chinese *Cobitis* species, which collectively formed a well-defined clade.

The interspecific genetic distance between *C. laterstriata* and *C. fimbriata* is 3.55%. The intraspecific genetic distances in *C. laterstriata*, *C. fimbriata*, *C. nigrolinea*, *C. qujiangensis*, *C. longibarba*, *C. brevipinna*, *C. xinjiangensis* and *C. delicata* are 0.69%, 0.88%, 0.65%, 0.47%, 0.09%, 1.16%, 1.09% and 0.65%, respectively (Table 4), which are much lower than the interspecific genetic distances.

**Table 4.** Genetic distances of the *Cytb* gene among ten species of *Cobitis*, which all lack sexual dimorphism.

		**Intraspecific**	**1**	**2**	**3**	**4**	**5**	**6**	**7**	**8**	**9**
1	*Cobitis laterstriata* sp. nov.	0.0069									
2	*Cobitis brevipinna*	0.0116	0.0607								
3	*Cobitis laterimaculata*	-	0.0518	0.0364							
4	*Cobitis fimbriata*	0.0088	0.0355	0.0612	0.0493						
5	*Cobitis nigrolinea*	0.0065	0.0398	0.0641	0.0481	0.0373					
6	*Cobitis qujiangensis*	0.0047	0.0442	0.0360	0.0250	0.0439	0.0418				
7	*Cobitis tigrina*	-	0.0462	0.0666	0.0531	0.0427	0.0436	0.0458			
8	*Cobitis longibarba*	0.0009	0.0512	0.0379	0.0228	0.0489	0.0464	0.0204	0.0518		
9	*Cobitis xinjiangensis*	0.0109	0.0434	0.0627	0.0529	0.0417	0.0455	0.0447	0.0448	0.0506	
10	*Cobitis delicata*	0.0065	0.1518	0.1649	0.1478	0.1473	0.1488	0.1513	0.1540	0.1432	0.1472

In conclusion, the molecular phylogenetic results support *C. laterstriata* as a distinct species.

## Discussion

**Figure 10.**
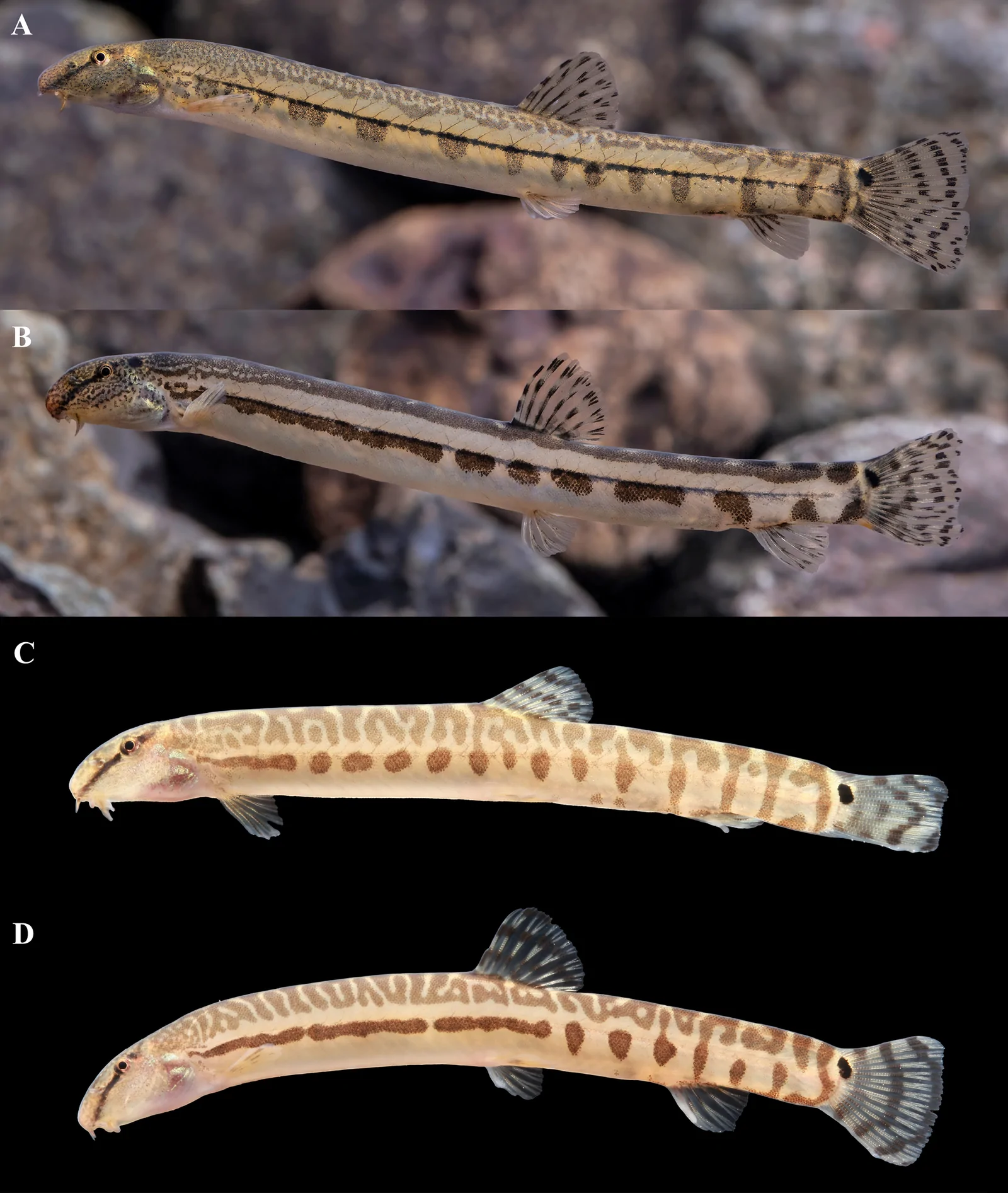
Two Chinese *Cobitis* species with similar color pattern. **A**. *Cobitis laterstriata* sp. nov., female; **B**. *Cobitis laterstriata* sp. nov., male; **C**. *Cobitis xinjiangensis*, female, ASIZB 254997, 54.9 mm standard length; **D**. *Cobitis xinjiangensis*, male, ASIZB 254996, 69.1 mm standard length.

*Cobitis laterstriata* can be clearly distinguished from the species lacking both sexual dimorphism and the Gambetta pattern in China by its special color pattern, especially the presence of a distinct black stripe running along the mid-lateral line of the body from the posterior edge of the operculum to the caudal-fin base—a feature that is absent in all other eight species. Notably, *C. laterstriata* is most similar to *C. xinjiangensis* in color pattern: blotches below the mid-lateral line are elongated and flattened in the anterior part of the body, and gradually become shorter and vertically oriented toward the posterior region, with a blank area above the mid-lateral line in both species. Moreover, both *C. xinjiangensis* and *C. laterstriata* exhibit a potentially sex-related color pattern: males predominantly exhibit fusion of the blotches below the mid-lateral line into a relatively long black stripe in the anterior body part, whereas females generally do not show such fusion (Fig. 10). Nevertheless, there are differences in color pattern between *C. laterstriata* and *C. xinjiangensis*: 1) conspicuous stripe with 14–19 small, short surface blotches on dorsum of *C. laterstriata* vs. an inconspicuous stripe with 17–20 large, long surface blotches on dorsum of *C. xinjiangensis*; 2) small and dense blotches on upper lateral side of *C. laterstriata* vs. broad vertical stripes continuous along dorsal blotches on upper lateral side of *C. xinjiangensis*; 3) blank area above mid-lateral line of *C. laterstriata* is larger; and 4) thin black stripe along mid-lateral line in *C. laterstriata* is more distinct and darker in color. However, it is difficult to distinguish these two species effectively solely based on their color pattern. *Cobitis laterstriata* can be further differentiated from *C. xinjiangensis* by the following diagnostic characters: 1) lower lip and mandible separated (vs. lower lip wrapped mandible); 2) short caudal peduncle, length 8.2–11.3% of SL (vs. 11.4–16.9%) (p < 0.001, U = 0.0); 3) possessing 19–20 pre-pelvic myotomes (vs. 17–18) (p < 0.001, U = 264); and 4) anal fin with four branched rays (vs. five) (Fig. 10).

Our molecular results show that *C. laterstriata* and *C. fimbriata* are sister species, and the monophyletic group formed by these two species is the sister group of *C. nigrolinea*. *Cobitis fimbriata* and *C. nigrolinea* are more morphologically similar to each other, but both exhibit significant morphological differences from *C. laterstriata*. In addition to the completely different body marking patterns, the following diagnostic differences are observed: 1) suborbital spine of *C. laterstriata* is curved (vs. straight in *C. fimbriata*) with a shorter lateral process (vs. longer lateral process in *C. nigrolinea*); 2) scales on the sub-dorsal region of *C. laterstriata* have a larger focal area (vs. smaller focal area in *C. fimbriata*); 3) *Cobitis laterstriata* has a shorter caudal peduncle, with caudal peduncle length accounting for 8.2–11.3% of the standard length (vs. 10.6–14.5% in *C. nigrolinea*, which has a longer caudal peduncle) (p < 0.001, U = 6.5); 4) number of pre-pelvic myomeres in *C. laterstriata* is 19–20 (vs. 17–18 in *C. nigrolinea*) (p < 0.001, U = 462); 5) six branched dorsal-fin rays in *C. laterstriata* (vs. seven in *C. fimbriata* and *C. nigrolinea*); and 6) four branched anal-fin rays in *C. laterstriata* (vs. five in *C. fimbriata* and *C. nigrolinea*) (Chen et al. 2017). Morphometric data of *C. laterstriata*, *C. xinjiangensis* and *C. nigrolinea* are presented in Table 2.

### Comparative materials

***Cobitis brevipinna***: nine specimens. ASIZB 255004, 50.9 mm SL; Tuankou Township, Linan District, Hangzhou City, Zhejiang Province, from the Tianmuxi River (30.0308°N, 119.1239°E). ASIZB 255005–255012, 41.7–49.0 mm SL; Dongdu Village, Jinyun County, Lishui City, Zhejiang Province, from the Oujiang River (28.6051°N, 120.0772°E); collected by Xiaofeng Yang.

***Cobitis nigrolinea***: 16 specimens. ASIZB 254876, 254965–254966, 254878–254880, 255019–255028, 51.7–78.3 mm SL; Liukou Township, Xiuning County, Huangshan City, Anhui Province, from the Xinanjiang River (29.7035°N, 117.7893°E).

***Cobitis xinjiangensis***: 16 specimens. ASIZB 254986–255001, 42.4–74.6 mm SL; Zihu Township, Yushan County, Shangrao City, Jiangxi Province, from the Xinjiang River (28.8935°N, 118.1593°E); collected by Zhixian Sun and Chongguang Chen.

## Supplementary Material

XML Treatment for Cobitis laterstriata

## Additional information

### Artificial Intelligence (AI) use

The authors accept full responsibility for the content of the manuscript, including the disclosure of any use of AI.

No AI tools were used in the preparation of this manuscript.

### Author ORCIDs

X.-Y. Li https://orcid.org/0009-0003-3379-1523

Z.-X. Sun https://orcid.org/0000-0003-0565-2801

Y.-T. Fang https://orcid.org/0009-0008-5347-9250

Y.-H. Zhao https://orcid.org/0000-0002-4615-596X

### Data availability

All of the data that support the findings of this study are available in the main text.
